# Combined spontaneous coronary artery dissection (SCAD) and Takotsubo syndrome (TTS): a case series

**DOI:** 10.1186/s43044-023-00361-6

**Published:** 2023-04-28

**Authors:** Saadat Ali Saleemi, Lung En Teng, Ronald J. L. Dick

**Affiliations:** 1 Department of Cardiology, Epworth Richmond, Victoria, 3121 Australia; 2 Department of General Medicine, Monash Health, Clayton, VIC 3168 Australia; 3 Department of General Medicine, Peninsula Health, Frankston, VIC 3199 Australia; 4 General Medicine/Cardiology AT, Monash Health, Clayton, VIC 3806 Australia

**Keywords:** SCAD, TTS, Cardiovascular, NSTEMI, Cardiomyopathy

## Abstract

**Background:**

Spontaneous Coronary Artery Dissection (SCAD) and Takotsubo Syndrome (TTS) are two different entities with several shared risk factors, but their management is different. They can co-exist in patients with chest pain which affects their management. We present two cases of combined SCAD and TTS in patients presented with chest pain.

**Case presentation:**

Case 1: 80F admitted with typical chest pain and dynamic ECG changes on the background of known anxiety/depression and social stresses. Her coronary angiogram showed SCAD affecting distal LAD. The left ventriculogram (LV gram) showed apical ballooning consistent with Takotsubo Syndrome (TTS). Patient was discharged on aspirin as well as angiotensin receptor blocker (ARB).

Case 2: 60F admitted with typical chest pain in the setting of emotional trauma on the background of known cardiovascular risk factors. She was found to have ST elevation in inferior leads with no reciprocal changes. Subsequently, coronary angiogram showed SCAD affecting mid-left anterior descending artery (LAD) with normal distal wrap around LAD. Her LV gram showed apical ballooning consistent with TTS. However, transthoracic echocardiogram showed akinetic left ventricular apex. She was discharged on aspirin as well as an ACE inhibitor and warfarin to prevent LV thrombus.

**Conclusions:**

SCAD and TTS can co-exist in patients with chest pain. It is important to identify SCAD in patients with TTS as it may affect their short as well as long-term management.

## Background

Spontaneous Coronary Artery Dissection (SCAD) is a rare cause of acute coronary syndrome with unknown incidence, whereas Takotsubo Syndrome (TTS) is a reversible apical ballooning syndrome. Both are associated with several physical, emotional and psychological risk factors. SCAD is managed with single or double antiplatelets therapy along with cardiovascular risk factor modifications, whereas TTS is a reversible cardiomyopathy. There are limited cases of combined SCAD and TTS in the literature and it is important to identify these cases as it may affect their ongoing management. We present two cases of combined SCAD and TTS with several shared risk factors.

## Case presentation

### Case 1

An 80 years old female referred by general practitioner (GP) with typical chest pain and dynamic ECG changes. Patient reported chest pain while visiting a local doctor. She also reported being significantly stressed in last couple of months after her husband was diagnosed with cancer. She was known to have paroxysmal atrial fibrillation (pAF) causing syncope and required dual chamber permanent pacemaker (PPM) in the past. Her other comorbidities included long-term anxiety/depression, gastroesophageal reflux disease (GORD) and hypercholesterolemia. Her usual medications included sotalol and rosuvastatin. She had been a lifelong non-smoker and only drank alcohol socially. Her main cardiovascular risk factor was significant family history of cardiac disease.

**Fig. 1:**
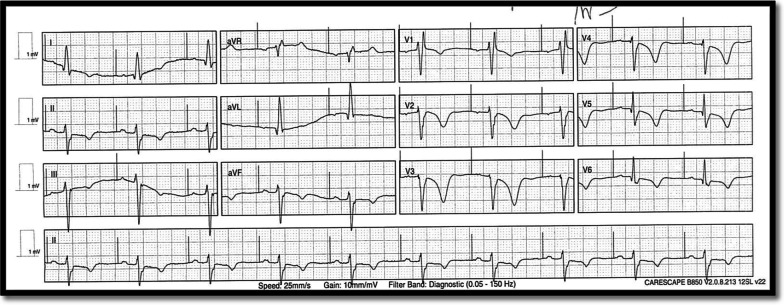
ECG of Case 1 showing paced rhythm, LAD, incomplete RBBB and deep TWI anterolateral leads

**Fig. 2:**
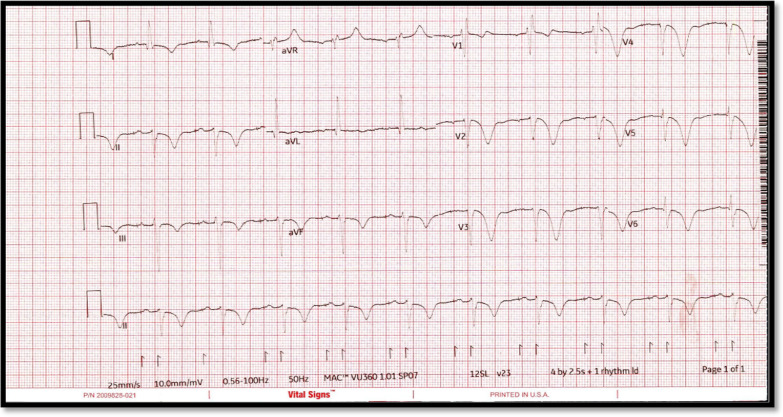
Another ECG of Case 1 showing paced rhythm, LAD and further deep TWI

On arrival to ED, she was hemodynamically stable with blood pressure of 130/70 and regular HR of 70 bpm. Her first ECG showed paced rhythm, left axis deviation (LAD), incomplete RBBB and deep TWI in anterolateral leads as shown in Fig. 1. Her T waves progressively deepened on later ECG as shown in Fig. 2.

Her baseline investigations including full blood exam and biochemistry were normal and chest X-ray showed dual chamber PPM with no signs of congestion. Her high sensitivity troponin T peaked at 1364 ng/L (normal < 15 ng/L). Her initial B- Natriuretic peptide (BNP) peaked at 419 pmol/L (normal < 35 pmol/L).

**Fig. 3:**
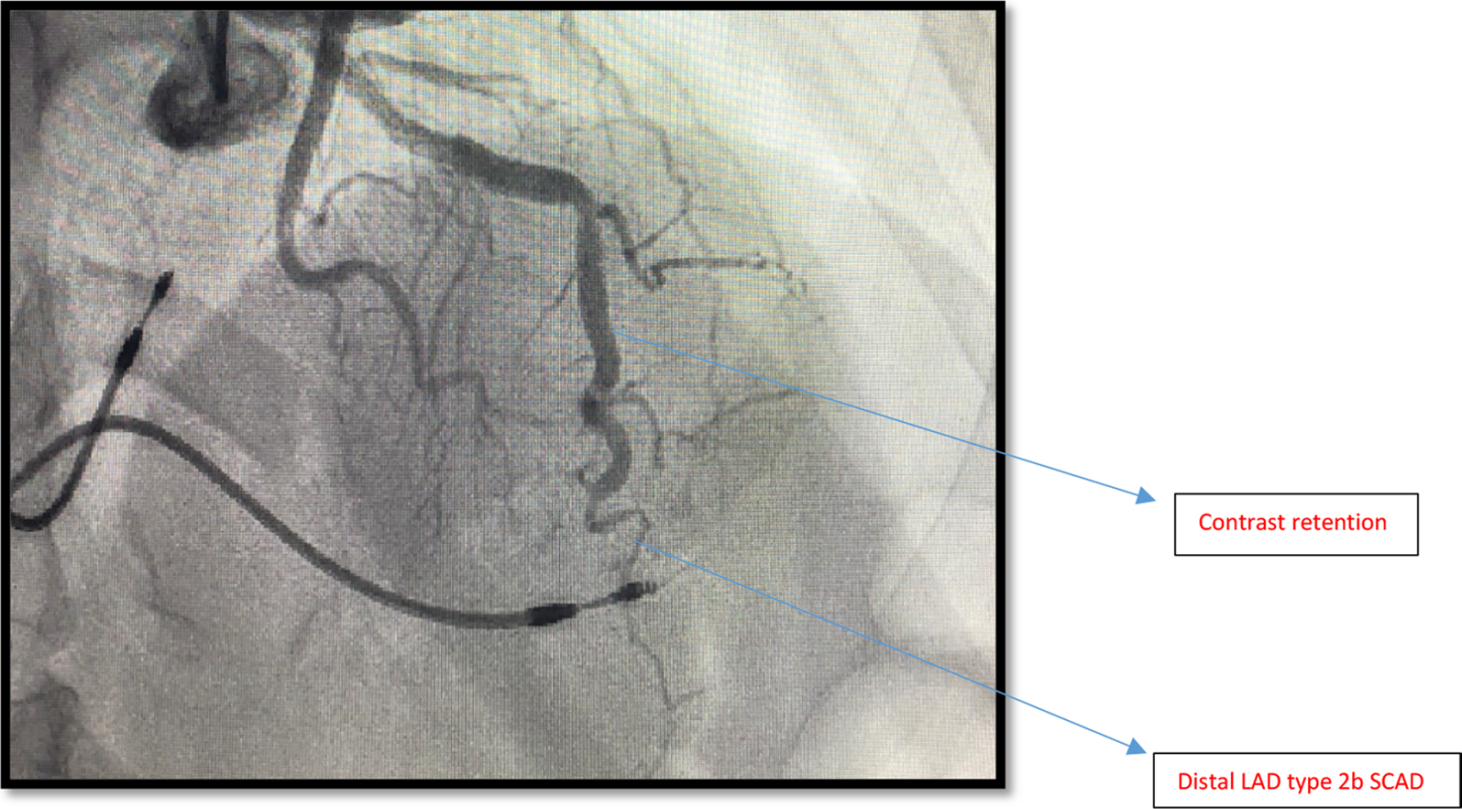
Mid LAD showing contrast retention and distal LAD SCAD—Case 1

**Fig. 4:**
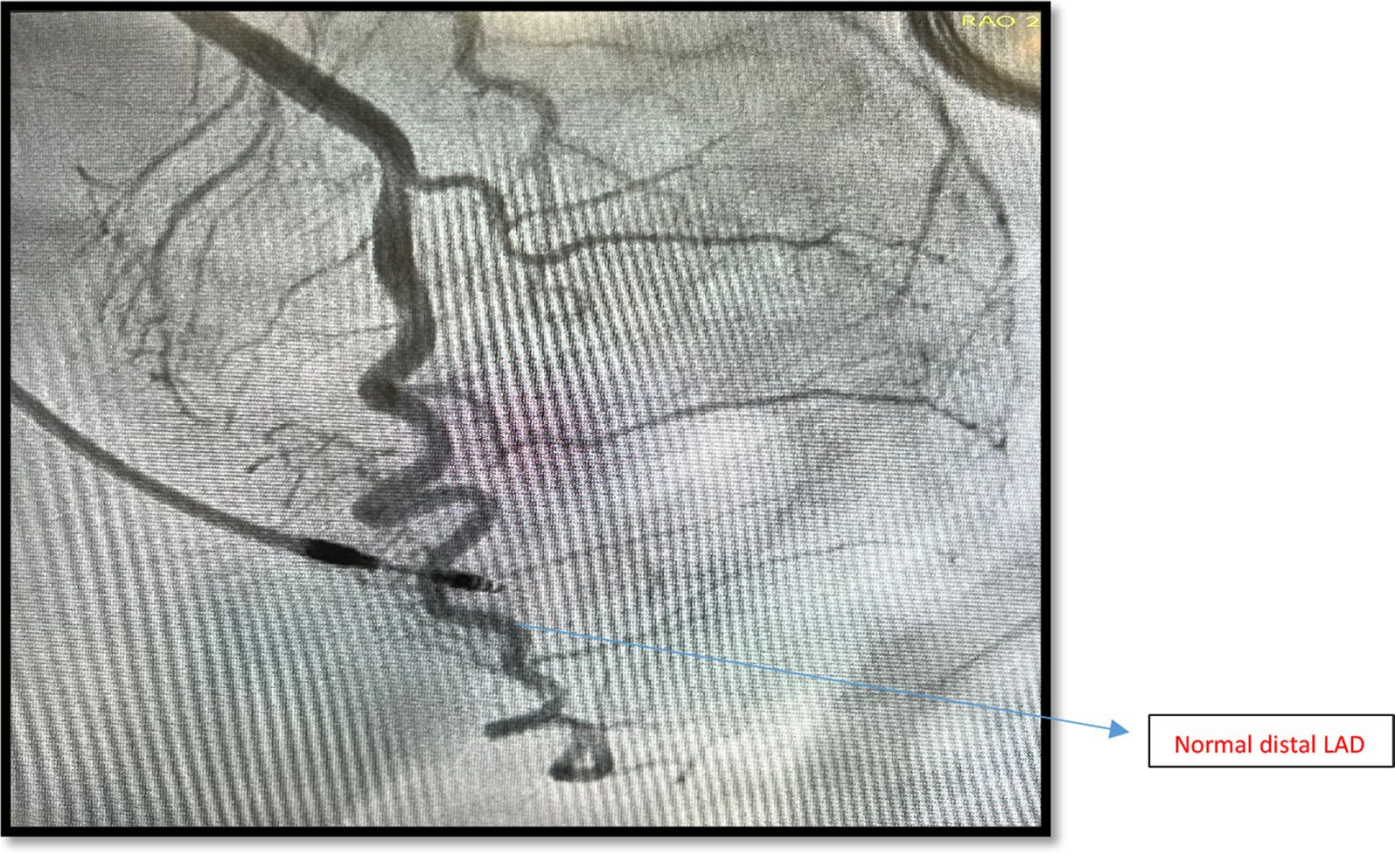
Old angiogram showing normal distal LAD—Case 1

**Fig. 5:**
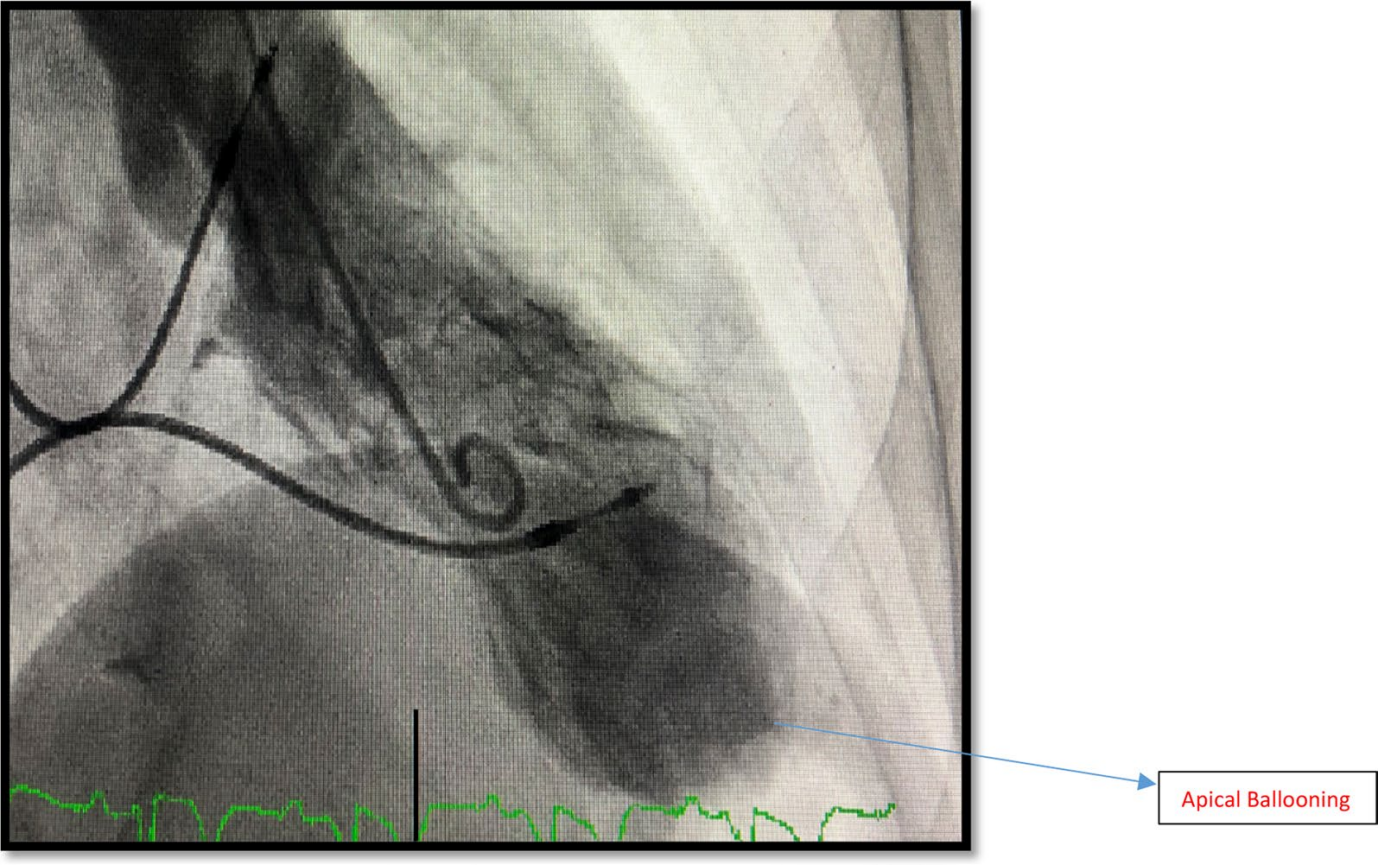
LV gram showing Apical Ballooning—Case 1

She proceeded to have a coronary angiogram to rule out coronary obstruction. Coronary angiogram showed 2 pathologies including non-obstructive coronary artery disease affecting left and right coronaries and distal left anterior descending (LAD) type 2 Spontaneous Coronary Aartery Dissection (SCAD) as shown in Fig. 3. Patient had another coronary angiogram one year ago. The distal LAD was found to be normal on it. (Fig. 4). Left ventriculography (LV gram) on our angiogram showed apical ballooning consistent with Takotsubo Syndrome (TTS) as shown in Fig. 5. She further had transthoracic echocardiogram showing dilated LV and moderate segmental systolic dysfunction. LV appearance was consistent with Takotsubo cardiomyopathy.

Patient remained inpatient for 4 days. She remained hemodynamically stable and assessed by private psychiatrist to manage the anxiety and started on Oxazepam. She was discharged on aspirin 100 mg daily along with her usual medications including rosuvastatin 5 mg daily and sotalol 80 mg BD. She was also started on angiotensin receptor blocker (ARB) given mild left ventricular dysfunction. Her 6 months followup transthoracic echocardiogram (TTE) showed recovered LV function consistent with Takotsubo Syndrome.

### Case 2

**Fig. 6:**
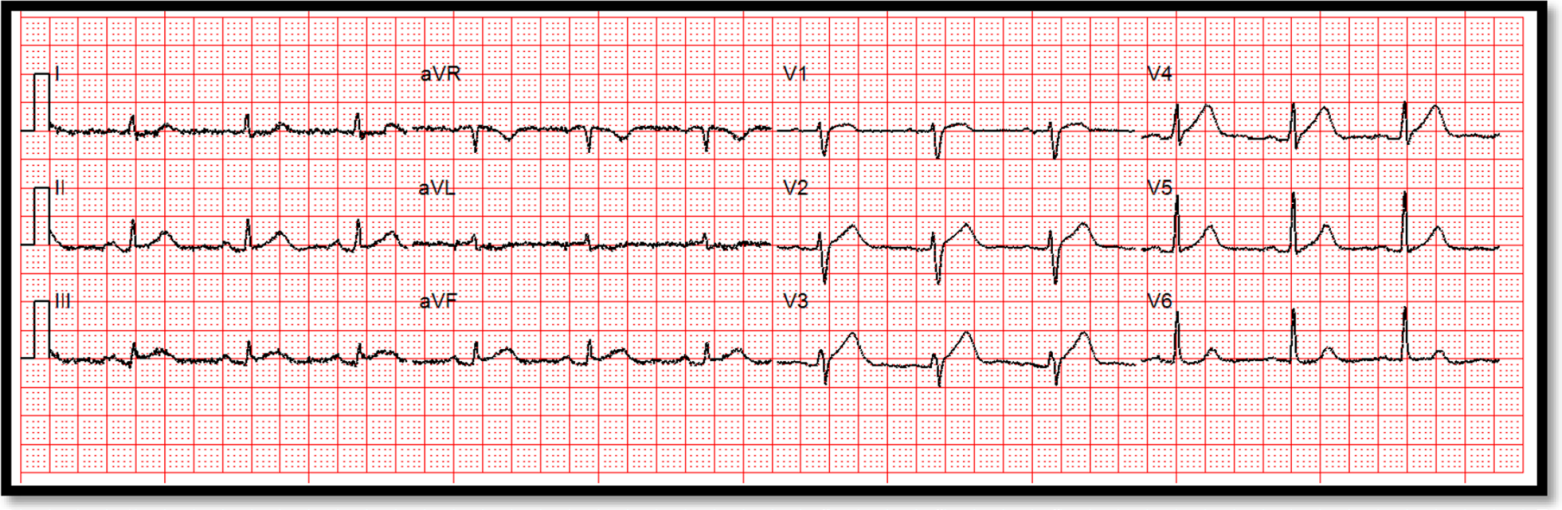
ECG on arrival to hospital—Courtesy Dr Riley Batchelor (RMH)

A 65 years old female presented to hospital with typical chest pain for further assessment. This is on the background of known cardiovascular risk factors including strong family history and previous smoking. She had no past medical history and was not on any medications. Her next of kin (NOK) was diagnosed with metastatic cancer. On the day of her presentation to hospital, her NOK was taken to hospital for further assessments and her chest pain coincided with that time. On her arrival to emergency department (ED), she was hemodynamically stable and her ECG showed SR, ST segment elevation in inferior leads (II, III, aVF) and no TWI in reciprocal leads (Fig. 6). Her baseline bloods were unremarkable and high sensitivity troponin T peaked at 9797 ng/L (normal < 15 ng/L).

**Fig. 7:**
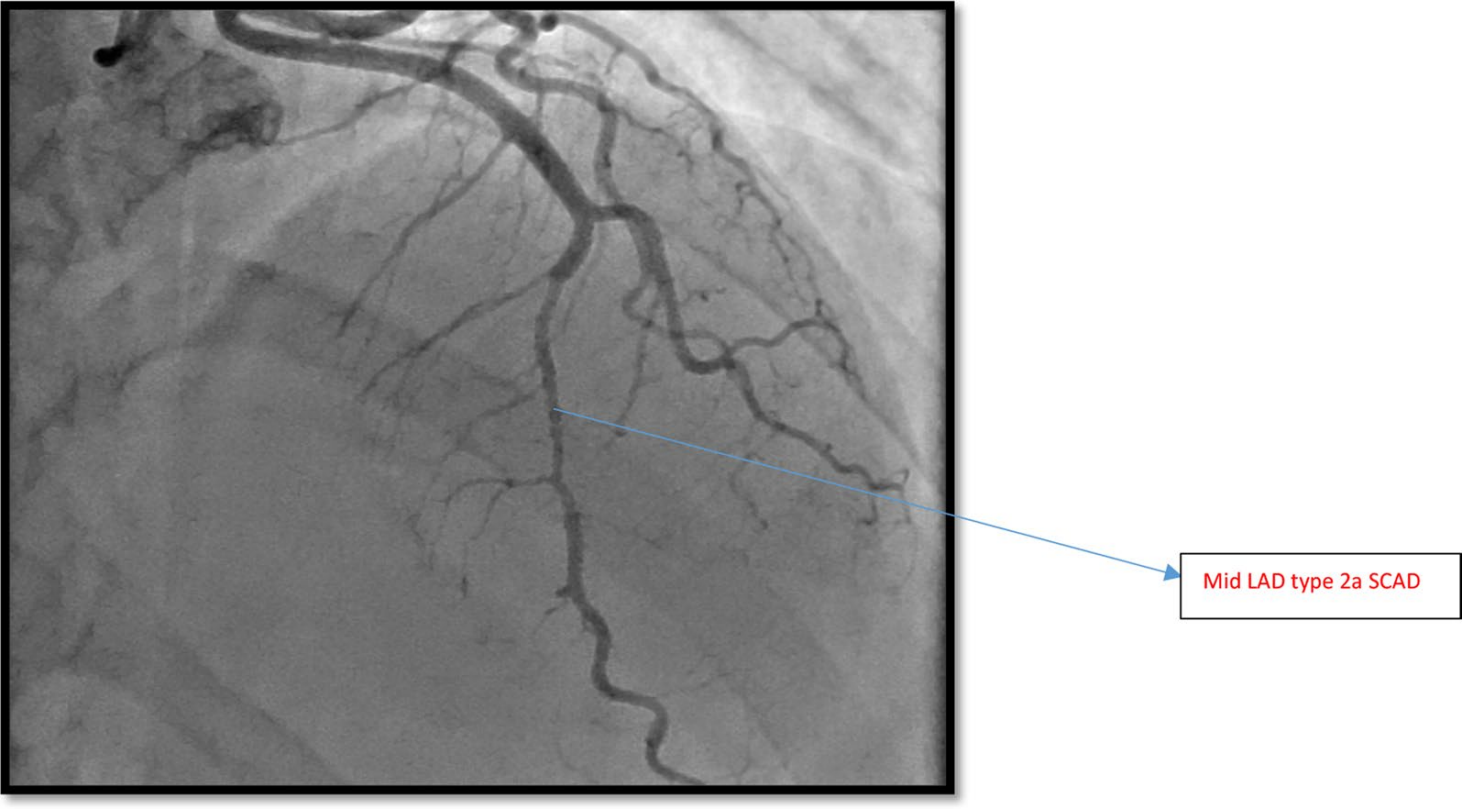
Mid LAD Type 2a SCAD—Case 2 (Courtesy Dr Riley Batchelor RMH)

**Fig. 8:**
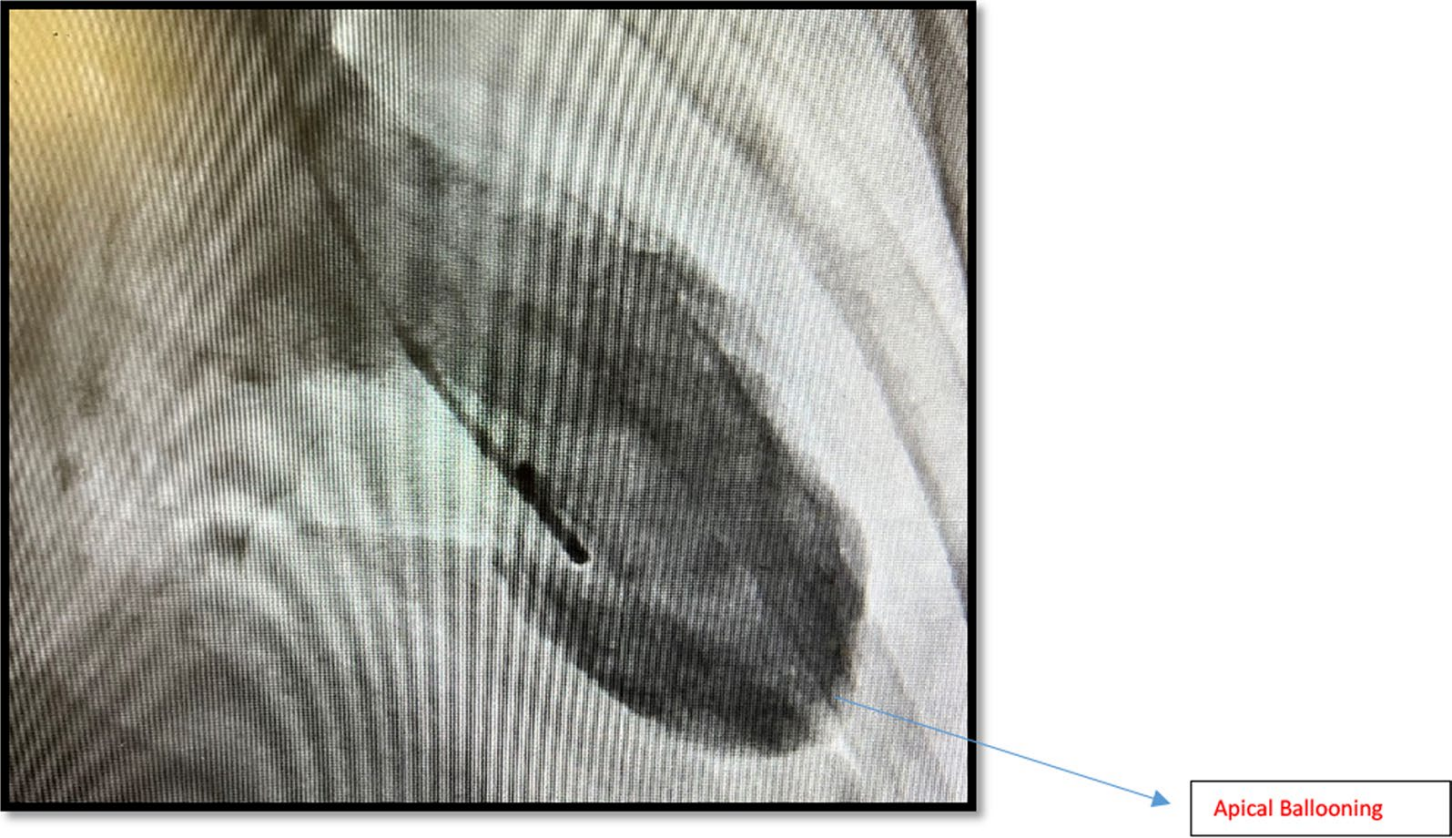
Apical Ballooning—Case 2—Courtesy Dr Riley Batchelor (RMH)

**Fig. 9:**
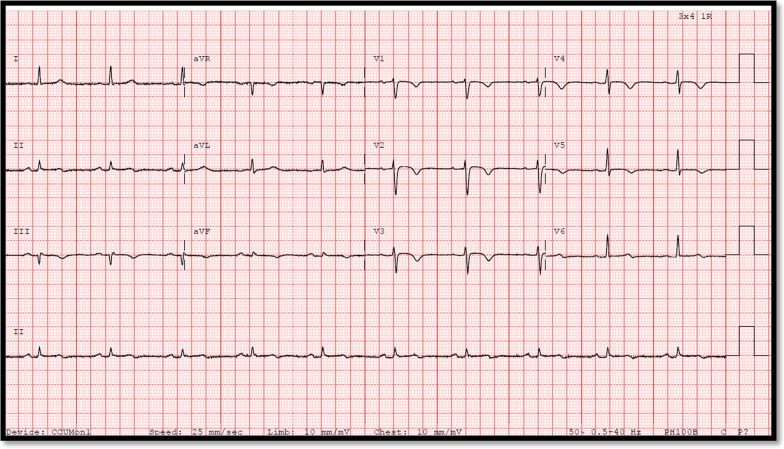
ECG showing TWI anteriorly post-coronary angiogram—Courtesy Dr Riley Batchelor (RMH)

She was taken to cardiac catheterization lab for coronary angiogram which showed normal right coronary artery (RCA) and mid-left anterior descending artery (LAD) had Spontaneous Coronary Artery Dissection (SCAD) with normal distal wrap around LAD as shown in Fig. 7. Her left ventriculography (LV gram) showed apical ballooning consistent with Takotsubo Syndrome as shown in Fig. 8. Post-coronary angiogram, her ECG showed TWI in anterior leads as shown in Fig. 9 and her transthoracic echocardiogram reported akinetic distal and apical segments which was consistent with LAD territory ischemia.

She had an uncomplicated admission and was discharged on aspirin 100 mg daily and atorvastatin 40 mg daily. She was also prescribed cardio-selective beta blocker and an ACE inhibitor for her LV dysfunction. Due to akinetic left ventricular apex, she was discharged on warfarin with bridging therapeutic clexane to prevent LV thrombus. Patient decided to be reviewed privately and lost the followup.

## Discussion

Spontaneous Coronary Artery Dissection (SCAD) is defined as the acute development of a false lumen within the coronary artery wall that leads to flow limitation by compressing the true lumen. It excludes iatrogenic coronary artery dissection, secondary to atherosclerosis and related to trauma [1]. It is relatively rare cause of acute coronary syndrome (ACS) and true incidence of SCAD is unknown but the prevalence of SCAD is reported as 4% [2]. Conversely, Takotsubo Syndrome (TTS) is also known as apical ballooning syndrome or Takotsubo cardiomyopathy or stress cardiomyopathy. It represents 1–2% of patients with ACS. The syndrome was first described in the Japanese population in 1991 and was given the name of ‘Tako-Tsubo’ after the round bottomed and narrow-necked Japanese fishing pot that resembles the typical shape of left ventricle systole during this condition. It is a reversible condition that is frequently precipitated by stressful event; hence, the history and clinical suspicion are of paramount importance [3].

SCAD mainly affects 81–96% of females as reported in three large SCAD series which is similar to TTS which affects > 90% post-menopausal female. Madhavan et al. reported emotional stressors (41%) being the most frequent cause of SCAD, followed by physical stressors (24%) [3]. SCAD is associated with several medical condition which are either predisposing factor or the precipitating factor. It includes fibromuscular dysplasia, collagen vascular disorder, chronic inflammatory systemic diseases, hypothyroidism, pregnancy and genetic [1, 2]. Coronary angiography is the gold standard to diagnose SCAD but in difficult cases intravascular ultrasound (IVUS) and optical coherence tomography (OCT) are required [1]. CT coronary angiography (CTCA) is potentially useful in diagnosing SCAD in acute settings where invasive procedure can be avoided as it may lead to secondary iatrogenic dissection but it is rather a clinical decision. However, CT coronary angiography’s role is rather limited in delayed setting as it tends to heal in few months.

There have been few case reports in recent times where SCAD and Takotsubo coexist [4, 5]. There are few hypotheses explaining their link together. It could be acute myocardial injury secondary to SCAD leads to myocardial damage and cascade of symptoms including acute chest pain and physical stresses which may result in Takotsubo syndrome. Case report 2 presented in a similar way with definite acute coronary event leads to Takotsubo syndrome. It could be a coincidence and might have been missed if LV gram was not performed. However, there are multiple risk factors which could precipitate both SCAD and TTS at the same time. There is also a concept of “adrenergic cardiomyopathy” whereby excessive circulating epinephrine causes cardiomyopathy by binding to beta 2 adrenergic receptors [6]. Salemi et al. reported a case of Takotsubo Cardiomyopathy in a patient with chronic obstructive pulmonary disease treated with inhaled beta 2 adrenergic agonist [7]. Similarly, Ioannou A et al. presented a case of iatrogenic adrenaline-induced mid-ventricular Takotsubo cardiomyopathy where patient was accidentally given intravenous adrenaline [8].

Management of both SCAD and TTS is different. Management of SCAD involves modification of cardiovascular risk factors with or without standard acute coronary syndrome (ACS) management. Cerrato et al. reported that dual antiplatelet therapy (DAPT) in the management of SCAD was independently associated with higher cardiovascular complication in comparison with single-agent antiplatelet therapy at 1 year [9]. However, TTS is considered as reversible cardiomyopathy and has a good prognosis. Hospital mortality associated with TTS is reported to be as low as 2% [3, 4].

## Conclusions

The similarities between SCAD and TTS presentation and causes can lead to missing the diagnosis and future management altogether. Therefore, it is extremely important to look closely for any SCAD in patients with the findings of apical ballooning on LV gram as it might change the treatment goals. Additionally, since SCAD is associated with several medical conditions hence knowing its existence might help identify those medical problems.

## Data Availability

Not applicable.

## Abbreviations

SCAD: Spontaneous coronary artery dissection
TC: Takotsubo syndrome
PPM: Permanent pacemaker
LAD: Left anterior descending
RCA: Right coronary artery
RBBB: Right bundle branch block
GORD: Gastroesophageal reflux disease
ARB: Angiotensin receptor blocker
ACS: Acute coronary syndrome
DAPT: Dual antiplatelet therapy
OCT: Optical coherence tomography
IVUS: Intravascular ultrasound
CTCA: CT Coronary angiogram
